# Introducing the special topic “The when and why of sensorimotor processes in conceptual knowledge and abstract concepts”

**DOI:** 10.3389/fnhum.2013.00498

**Published:** 2013-08-26

**Authors:** Barbara Tomasino, Raffaella I. Rumiati

**Affiliations:** ^1^I.R.C.C.S. “E. Medea”, Polo Friuli Venezia GiuliaSan Vito al Tagliamento, Italy; ^2^Neuroscience Area, SISSATrieste, Italy

**Keywords:** actionrepresentations, motorsystem, wordmeaning, sensorimotor cortex, action verbs

A fundamental question of cognitive neuroscience concerns the role of sensory and motor information in representing the conceptual knowledge in the brain. Indeed, the extent to which conceptual representations are held to be grounded in sensory and motor systems has yielded different hypotheses as to how conceptual knowledge is organized. On the one hand, the embodied hypothesis promotes the idea that conceptual representations are modality-dependent and built from sensory and motor experiences, that is by re-enacting sensorimotor memories acquired through experience (Barsalou, 1999; Pulvermuller et al., 1999; Barsalou et al., 2003; Gallese and Lakoff, 2005). Thus, recognizing objects, actions and words is accomplished by re-enacting sensorimotor memories that have been previously acquired (this is also called motor simulation). On the opposite extreme, the disembodied hypothesis holds that conceptual representations are abstract (symbolic) and modality-independent (amodal), separated from sensorimotor information, e.g., (Fodor, 1983; Caramazza et al., 1990; Tyler and Moss, 2001). To reconcile these two extreme views, the grounding by interaction hypothesis proposes that what we know about words, for instance, is meant to benefit from the contribution of both abstract content and sensory and motor systems (Mahon and Caramazza, 2008; Bedny and Caramazza, 2011).

From the beginning, neuropsychological and neuroimaging studies contributed to this debate with the necessary evidence to constrain hypotheses about the role of sensory and motor systems in understanding objects, actions and words. The three theoretical accounts reviewed above generate different predictions as to the involvement of such systems in these cognitive operations. For the embodied hypothesis, the involvement of sensorimotor systems appears to be a fundamental, however, how the brain implements abstract concepts and symbolic operations is still not easily explained within the embodied account. According to the disembodied hypothesis the involvement of mental simulation is ancillary, whereas the grounding by interaction hypothesis specifies its dependency upon the contextual factors. Even though both the disembodied hypothesis and grounding by interaction hypothesis agree on concepts being stored in an abstract way, a direct demonstration that this is actually the case is seldom documented. A related aspect that still requires more theoretical and empirical effort concerns the role of implicit motor imagery in understanding words. In fact, despite the growing evidence, results are contradictory: motor activity has been observed not only for action-related verbs but also for imaginable concrete words that are not grounded in sensorimotor experience.

In order to promote the development of the neuroscientific investigation and discussion on how conceptual knowledge is represented, this Frontiers Research Topic aimed at bringing together contributions from researchers whose interests focus on the action-related and abstract concepts processing. We collected both reviews and original research articles in which the authors used neuropsychology, behavioral methods, electromyography recordings, event-related potentials, fMRI experiments on patients and healthy controls, and reversible virtual lesions. Taken together these contributions strongly indicate that the role of the sensorimotor context is neither automatic nor a necessary one.

In a study in which the neuropsychological approach was used, Gvion and Friedmann (2013) presented the intriguing case of patient Nissim with a lesion of the left occipital lobe whose ability to retrieve and understand words with visual and sensory characteristics, such as ball, spoon, carrot (and proper names) was dependent on the item imageability. The patient showed severe difficulties in retrieving and understanding imageable words, while with abstract and complex items he was perfect. Nissim's ability to retrieve gestures for objects and pictures he saw was much better than his retrieval of the names of the same objects. Kemmerer et al. (2013) studied 10 patients with Parkinson's disease who performed a semantic judgment task including action and non-action related verbs both while they were ON and OFF medication as accurately as a group of 10 healthy controls. Garcea et al. (2013) studied patient AA with a left fronto-parietal lesion and hemiplegia who presented a dissociation between action and object knowledge, with an impairment in object-associated action production and in his conceptual knowledge about actions, while his knowledge of objects was largely preserved. Maieron et al. (2013), combining neuropsychological and fMRI-PPI connectivity data, failed to find an effect of neurosurgical lesions in the primary motor cortex (M1) on the ability to name action verbs as well as a functional coupling between M1 and functional nodes of the linguistic network during verb generation for both controls and patients. Crutch et al. (2013) used a new approach, i.e., the abstract cognitive feature (ACF), to examine semantic relatedness of abstract words and to obtain ratings of the contribution of different cognitive systems (e.g., sensation, action, emotion morality, space, time, social interaction) to abstract concepts. The mapping was tested and confirmed by studying patient SKO, with a lesion involving the left fronto-parietal area causing him a verbal comprehension deficit, who was significantly worse at distinguishing targets presented within word pairs with low ACF distances. Items with small distance are more semantically related and therefore more difficult to distinguish for a patient with impaired comprehension.

In a study based on reversible virtual lesions produced by transcranial magnetic stimulation (TMS), Sartori et al. (2013) stimulated M1 while left- and right-handed participants observed a left- or a right-handed model grasping an object. The authors found that motor resonance is mediated by effector-independent motor representations, since the observer's handedness shaped motor resonance in right- as well as in left-handers regardless of the identity of the observed hand, and the correspondence between the model's and the observer's effector was no longer revealed in the non-dominant hand.

Putting a cognitive network under stress can be a way to simulate neuropsychological deficits, e.g., (Tessari and Rumiati, 2004). Postle et al. (2013) used a dual task paradigm, where concurrent processing of hand related information should interfere more with hand tapping movements than processing of unrelated body parts (e.g., foot or mouth actions) information. Concurrent reading of single words related to specific body-parts, or the same words embedded in sentences differing in syntactic and phonological complexity (to manipulate context-relevant processing), and reading while viewing videos of the actions and body-parts described by the target words (to elicit visuomotor associations) all interfered with the right-hand but not left-hand tapping rate. However, this motor interference was not differentially affected by hand-related stimuli. Thus, the results provide no support for proposals that body-part specific resources in cortical motor systems are shared between overt manual movements and meaning-related processing of words related to the hand. In another behavioral study, Cacciari and Pesciarelli (2013) investigated the relation between the non-literal use of language and the sensorimotor activation by showing that foot button presses were significantly faster than finger responses only for foot-related actions embedded in literal motion, as compared to fictive, idiomatic, metaphorical motion related items, thus confirming that the sensorimotor activation in linguistic processing is constrained by the linguistic context in which stimuli occur.

Taking advantage of electromyography (EMG) recordings, Foroni and Semin (2013) showed that the response of the muscles involved in the description of an action is non-automatic but rather modulated by the context. A context-dependent activation of the zygomatic muscle while processing sentences describing emotional expressions was found while the negation forms of these sentences inhibited zygomatic muscle activity as measured by EMG, as compared to when the same sentences were presented in an affirmative form.

Studies using fMRI also evidenced that the sensorimotor activation is not solely triggered bottom-up by action word stimuli. Schuil et al. (2013) showed that the activation of motor regions is context-dependent and it is greater for silent reading of arm and leg related actions presented in a literal context than for non-literal contexts. However, this was independent of stimulus category, i.e., there was no evidence for a semantic somatotopic organization of the motor cortex. In addition, Sakreida et al. (2013) found sensorimotor cortex activation for silent reading of both concrete and abstract multi-word expressions in an action context. Eckers et al. (2013) showed that syllable processing activated the precentral gyrus bilaterally, independent of the input modality and response mode, supporting the existence of a supramodal hub and different sensorimotor representations. They provided preliminary evidence for the speech-action-repository or mental syllabary as the central module for sensorimotor processing of syllables. Lastly, Kumar et al. (2013) used *mu rhythm* analysis over regions involved in motor programming and enactment and showed that motor-based affordances such as hand grips (irrelevant to the task) affected object recognition, thus confirming a tight interaction between the action and object recognition domains often acknowledged in recent years.

In addition to original research articles, the present special topic includes also reviews as well as hypothesis and theory articles. Papeo et al. (2013) reviewed TMS studies in which lexical-semantic tasks have been used as paradigms, and words as stimuli. They showed that TMS induced effects on the M1 and the premotor cortex cause behavioral changes that are inconsistent and thus argued that the relation between action word processing and the motor system is far from clear. Amoruso et al. (2013), on the other hand, reviewed the literature on the N400 component, considered a neural signature of the semantic integration of a given stimulus into a previous context, and showed that it is involved in the processing of meaning based on the expectancies formed by previous experiences and that it is highly context-dependent. Crepaldi et al. (2013) carried out a meta-analysis on neuroimaging data of noun and verbs processing by using hierarchical clustering algorithm, and concluded that there is no evidence in support of the view that verbs processing is based on embodied motoric information.

The last contribution of this special issue is by Shallice and Cooper (2013) who argued that the embodied view and the feature-based representation of semantics are insufficiently powerful to capture abstract concepts-related processing. In addition, patients with reversed concreteness effect and those with deep dyslexia are reviewed as some evidence that the semantic representations of abstract and concrete words are separable in the cognitive system. This view is supported from the fMRI studies which highlight the importance of the inferior frontal gyrus in processing abstract-related words.

Taken together, these studies indicate that sensorimotor activation is not automatically triggered by the type of stimulus and it is not necessary but accessory to linguistic processing (Mahon and Caramazza, 2005, 2008; Papeo et al., 2009; Raposo et al., 2009; Tomasino et al., 2010a,b; Willems et al., 2010; Postle et al., 2013). Rather, results indicate that the involvement of sensorimotor areas depends on the context (van Dam et al., 2010, 2012a,b) in which conceptual features are retrieved. Flexibility is characterized by the relative presence or absence of activation in motor and perceptual brain areas. In addition, the involvement of sensorimotor areas may be subject to a top-down modulation which explicitly or automatically select the type of strategy adopted while processing language (Tomasino and Rumiati, 2013).
